# Hyperphosphatasia with mental retardation syndrome 3: Cerebrospinal fluid abnormalities and correction with pyridoxine and Folinic acid

**DOI:** 10.1002/jmd2.12347

**Published:** 2022-11-22

**Authors:** Martina Messina, Emanuela Manea, Thomas Cullup, Karin Tuschl, Spyros Batzios

**Affiliations:** ^1^ Metabolic Medicine Department Great Ormond Street Hospital for Children London UK; ^2^ North Thames Genomic Laboratory Hub Great Ormond Street Hospital for Children London UK; ^3^ University College London Great Ormond Street Institute for Children London UK

**Keywords:** CSF, developmental delay, folinic acid, GPI‐anchor defects, HPMRS, pyridoxine, PGAP2‐CDG

## Abstract

Glycosylphosphatidylinositol anchored proteins (GPI‐APs) represent a class of molecules attached to the external leaflet of the plasma membrane by the GPI anchor where they play important roles in numerous cellular processes including neurogenesis, cell adhesion, immune response and signalling. Within the group of GPI anchor defects, six present with the clinical phenotype of Hyperphosphatasia with Mental Retardation Syndrome (HPMRS, Mabry Syndrome) characterized by moderate to severe intellectual disability, dysmorphic features, hypotonia, seizures and persistent hyperphosphatasia. We report the case of a 5‐year‐old female with global developmental delay associated with precocious puberty and persistently raised plasma alkaline phosphatase. Targeted next generation sequencing analysis of the HPMRS genes identified novel compound heterozygous variants in the *PGAP2* gene (c.103del p.(Leu35Serfs*90)and c.134A > Gp.(His45Arg)) consistent with the diagnosis of HPMRS type 3. Cerebrospinal fluid (CSF) neurotransmitter analysis showed low levels of pyridoxal phosphate and 5‐methyltetrahydrofolate and raised homovanillic acid. Supplementation with pyridoxine and folinic acid led to normalization of biochemical abnormalities. The patient continues to make developmental progress with significant improvement in speech and fine motor skills. Our reported case expands the clinical spectrum of HPMRS3 in which multisystem involvement is being increasingly recognized. Furthermore, it shows that miss‐targeting GPI‐APs and the effect on normal cellular function could provide a physiopathologic explanation for the CSF biochemical abnormalities with management implications for a group of disorders that currently has no treatment that can lead possibly to improved clinical outcomes.

**Synopsis**

This article describes a new case of an HPMRS patient providing novel insights into symptomatology and disease management with pyridoxine and folinic acid supplementation.

## 1 INTRODUCTION

The glycosylphosphatidylinositols (GPI) are complex glycolipids synthesized in different cell types and anchoring more than 250 proteins (0.5% of all cell surface proteins) to the plasma membrane.^1^

At least 26 genes are involved in the biosynthesis of GPI‐anchored proteins (GPI‐APs).^2^ The biosynthesis of the GPI anchor itself requires the co‐operative action of more than 18 proteins.^1^

Mutations in the genes involved in this process result in Glycosylphosphatidylinositol biosynthesis defects (GPIBDs) and are associated with broad clinical features.^2^, ^3^ To date, abnormalities in this pathway are believed to be the cause of several disorders among which hyperphosphatasia with mental retardation syndrome (HPMRS).^4^

HPMRS, first described by Mabry in 1970 and hence termed Mabry syndrome,^3^ comprises a group of autosomal recessively inherited disorders characterized by persistent hyperphosphatasia and moderate to severe postnatal neurodegenerative disorder with seizures and cognitive deficit.^1^ Six genes, and consequent enzymatic defects, are responsible for the six subtypes of HPMRS: *PIGV*, encoding alpha 1–6 mannosyltransferase II, *PIGO*, an EtNP transferase III, *PIGW*, the inositol acyltransferase, *PIGY*, a GPI‐GlcNAc transferase, involved in structuring the GPI anchor, and *PGAP2* and *PGAP3*, involved in the post‐translational modification after attachment of protein and GPI anchor.^1^, ^2^, ^4^, ^5^, ^6^, ^7^, ^8^, ^9^, ^10^, ^11^

The phenotypic spectrum is heterogenous and keeps expanding as new cases are described in literature. After normal perinatal period and unremarkable development and growth during the first months of life, progressive developmental delay, intellectual disability and, in most cases, seizures and epilepsy present.^1^ An overview of clinical findings for all subtypes is presented in Table 1.

**TABLE 1:** HPMRS phenotype overview, listed by subtypes (HPMRS 1–6)

HPMRS type	1	2	3	4	5	6
Gene	PIGV^7^, ^23^, ^24^, ^25^	PIGO^2^, ^4^, ^5^, ^9^, ^10^, ^17^, ^26^, ^27^, ^28^	PGAP2^8^, ^12^, ^14^, ^15^, ^16^	PGAP3^29^, ^30^, ^31^, ^32^, ^33^, ^34^, ^35^, ^36^, ^37^, ^38^	PIGW^6^, ^39^, ^40^	PIGY^41^
Inheritance	AR	AR	AR	AR	AR	AR
Growth	normal	NA – data limited	normal	normal	normal – few data reported	abnormal – poor growth
Signs and Symptoms						
Neurology						
*Intellectual disability*	+	++	++	++	++	++
*Developmental delay*	++	++	++	++	++	++
*Hypotonia*	+	++	+	++	++	−
*Seizures/epilepsy*	+	++	++	++	++	+
Neurosensorial						
*Hearing impairment*	+/−	+	+	+	−	−
*Vision impairment*	NA	NA	+	NA	NA	+ (congenital cataract, strabism)
Cardiological abnormalities	+/−	+/−	+/−	+	−	−
Gastrointestinal/anorectal abnormalities	+ (Hirschprung, anterior anus, abdominal distension)	+ (anal stenosis, anal atresia, mild anal prolapse, inguinal hernia)	+/−	+/− (inguinal hernia, constipation)	−	+/− (poor feeding, abdominal distension, NEC)
Vescicouretral anomalies	+	+/−(hypogenitalism)	−	+/−	−	+/−
Endocrine dysfunction	−	−	precocious adrenarche	−	−	−
Facial dysmorphism						
*Broad nasal bridge*	++	+/−	+/−	++	+	−
*Tented up lips*	++	+	+/−	++	+	−
*Hypertelorism*	++	+/−	+/−	++	−	+/−
*Wide palpebral fissures*			+/−			
Cleft palate	+	+	+/−	++	−	−
Abnormalities of extremities	++ (brachytelephalangyhypoplastic finger and toenails, hypoplastic distal phalange)	++ (brachytelephalangyhypoplastic finger and toenails, hypoplastic distal phalange)	+/−	+/−	−	+ (brachytelephalangy)
Investigations						
ALP	++	++	+	++	+	+
Brain imaging	Mostly normal ‐ one reported poorly formed cerebellar vermis	Abnormal – hypoplasia of cerebellar vermis and brainstem, hypomyelination and abnormal T1 and T2 signal in basal ganglia to brainstem, increased DWI signal in globus pallidus and dorsal brainstem, signs of cerebral atrophy	Mostly normal – signs of cerebral atrophy, hypoplasia of the corpus callosum reported, Dandy Walker malformation	Abnormal – thin corpus callosum, hypoplasia of cerebellar vermis, dilated ventricles, brain atrophy, fronto‐parietal atrophy, mega cisterna magna, small capsula interna, Dandy‐Walker variant	Abnormal ‐ widened subarachnoid space	Abnormal ‐ white matter loss
CSF study	NA	NA	pyridoxine 8 mmol/L (ref. 10–37); 5MTHF 66 mmol/L (ref. 72–172)	NA	Investigated for infection, no Neurotransmitter done	NA

Hyperphosphatasia with mental retardation syndrome type 3 is caused by a defect in the *PGAP2* gene encoding the lipid remodelling step of GPI‐ anchor maturation and is required for stable expression of GPI‐APs on the cell surface.^11^ In PGAP2‐deficient cells, GPI‐APs, that contain the glycan but lack the lipid moiety GPIs, are prematurely released after transport to the cell surface.^12^, ^13^ PGAP2 is highly expressed in the brain, skeletal muscle, heart, and foetal liver consistent with the disease phenotype and organ involvement.^14^ The clinical phenotype is characterized by varying degrees of developmental delay, intellectual disability and elevated ALP, but unlike other HPMRS types, epilepsy, hypotonia, dysmorphisms and organ anomalies are less common (Table 3).^8^, ^11^, ^12^, ^14^, ^15^, ^16^ Including our patient, 20 individuals with homozygous or compound heterozygous missense variants have been described to date (Table 2).^8^, ^12^, ^14^, ^15^, ^16^ Their clinical characteristics are described in Table 3.

**TABLE 2:** PGAP2 variants reported in HPMRS3 patients

S. no	Variant^a^	Consequence	State	Origin	Reference
1	c.296A > G	p.Tyr99Cys	Homozygous	Syrian	Hansen et al.^8^
2	c.530G > C	p.Arg177Pro	Homozygous	Pakistani	Hansen et al.^8^
3	c.46C > T c.479C > T	p.Arg16Trp p.Thr160Ile	Heterozygous	Finnish	Krawitz et al.^12^
4	c.380 T > C	p.Leu127Ser	Homozygous	Turkish	Krawitz et al.^12^
5	c.191C > T^b^	p. Ala64Val	Homozygous	Saudi Arabia	Naseer et al.^16^
6	c.2 T > G c.221G > A	p.? p.Arg74His	Heterozygous	Polish	Jezela‐Stanek et al.^15^
7	c.554G > A	p.His185Gln	Homozygous	Bedouin	Perez et al.^14^
8	c.[698C > T]	p.Thr233Met	Homozygous	NA	Thompson et al.^42^
9	c.103del c.134A > G	p.Leu35Serfs*90p.His45Arg	Heterozygous	Afro‐Carribean	Present study

^a^ Variants listed on transcript NM_001256240.2 other than c.191C > T from Naseer et al. (see [2]).

^b^ Annotated on transcript NM_014489.4.

**TABLE 3:** Clinical findings in HPMRS 3 patients

Paper	Present study	Hansen et al.^8^							Krawitz et al.^12^	
Patients	1	1	2	3	4	5	6	7	1	2
Families	Family 1	Family 2			Family 3				Family 4	Family 5
Gender	female	Female	Female	Female	NA	NA	NA	NA	Female	Male
Ethinicity	afro‐carribean	Syrian	Syrian	Syrian	Pakistani	Pakistani	Pakistani	Pakistani	Finnish	Turkish
Consanguineuity	No	NA	NA	NA	NA	NA	NA	NA	No	Yes
Growth	enhanced	Normal	Normal	Normal	Normal	Normal	Normal	Normal	Normal	Normal
Signs and Symptoms										
Neurology										
*Intellectual disability*	−	+	+	+	+	+	+	+	mild	+
*Developmental delay*	−	+	+	+	+	+	+	+	mild	+
*Hypotonia*	+	+	+	+	NA	NA	NA	NA	−	+
*Seizures/epilepsy*	−	−	−	+	−	−	−	−	+ (valproate)	+
Neurosensorial										
*Hearing impairment*	−	−	−	−	−	−	−	−	−	+
*Vision impairment*	Esotropia, myopic astrigmatis	Strabism	Strabism	−	−	−	−	−	−	−
Cardiological abnormalities	−	−	−	−	−	−	−	−	−	ASD
Gastrointestinal/anorectal abnormalities	−	−	−	−	−	−	−	−		Hirschprung
Vescicouretral anomalies	−	−	−	−	−	−	−	−	−	−
Endocrine disfunction	Precocious adrenarche	NA	−	−	−	−	−	−	−	−
Facial dysmorphism										
*Broad nasal bridge*	−	−	−	−	−	−	−	−	+	+
*Tented up lips*	−	−	−	−	−	−	−	−	+	+
*Hypertelorism*	−	−	−	−	−	−	−	−	−	+
*Wide palpebral fissures*	−	−	−	−	−	−	−	−	−	+
Cleft palate	−	−	−	−	−	−	−	−	−	+
Extremities abnormalities	−	−	−	−	−	−	−	−	−	Broad fingernails and bilateral hypoplastic fifth fingernail
Investigations										
ALP	High	High	High	NA	NA	NA	NA	NA	High	High
Brain imaging	Normal (MRI brain)	Atrophy and increased gyration (ct head)	Atrophy and increased gyration (ct head)	Atrophy (CT head)	Normal (MRI brain)	Normal (MRI brain)	Normal (MRI brain)	Normal (MRI brain)	NA	Hypoplasia of the corpus callosum (CT head)
CSF study	pyridoxine 8 mmol/L (ref. 10–37); 5MTHF 66 mmol/L (ref. 72–172)	NA	NA	NA	NA	NA	NA	NA	NA	NA
Genetics										
Variants^a^	c.[103del]; [134A > G]	c.[296A > G];[296A > G]	c.[296A > G];[296A > G]	c.[296A > G];[296A > G]	c.[530G > C];[530G > C]	c.[530G > C];[530G > C]	c.[530G > C];[530G > C]	c.[530G > C];[530G > C]	c.[46C > T];[479C > T]	c.[380 T > C]; [380 T > C]

Paper	Naseer et al.^16^		Jezela‐Stanek et al.^15^	Perez et al.^14^				Thompson et al.^42^		
Patients	1	2	1	1	2	3	4	1	2	3
Families	Family 6		Family 7	Family 8				Family 9		Family 10
Gender	Male	Female	Female	Male	Male	Female	Female	Male	Male	Male
Ethinicity	Saudi Arabia	Saudi Arabia	Polish	Bedouin	Bedouin	Bedouin	Bedouin	NA	NA	NA
Consanguineuity	Yes	Yes	No	Yes	Yes	Yes	Yes	Yes	Yes	Yes
Growth	Normal (microcephaly)	Normal (microcephaly)	Normal	NA	NA	NA	NA	NA	NA	NA
Signs and Symptoms										
Neurology										
*Intellectual disability*	+	+	+	mild	+	+	mild	+	+	NA
*Developmental delay*	+	+	+	mild	+	mild	+	+	+	+
*Hypotonia*	NA	NA	+	NA	NA	NA	NA	+	+	
*Seizures/epilepsy*	+	+	+	+	+	−	−	+	+	+
Neurosensorial										
*Hearing impairment*	+	−	NA	−	−	−	−	NA	NA	NA
*Vision impairment*	−	+	NA	−	−	−	−	NA	NA	NA
Cardiological abnormalities	−	−	−	−	−	−	−	NA	NA	NA
Gastrointestinal/anorectal abnormalities	−	−	−	−	−	−	−	Normal rectal biopsy	Normal rectal biopsy	NA
Vescicouretral anomalies	−	−	−	−	−	−	Enuresis	NA	NA	NA
Endocrine disfunction	−	−	−	−	−	−	−	NA	NA	N A
Facial dysmorphism										
*Broad nasal bridge*	−	−	−	−	−	−	−	+	+	+
*Tented up lips*	−	−	−	−	−	−	−	+	+	+
*Hypertelorism*	−	−	−	−	−	−	−	−	−	−
*Wide palpebral fissures*	−	−	−	−	−	−	−	−	−	−
Cleft palate	−	−	−	−	−	−	−	NA	NA	NA
Extremities abnormalities	−	−	Pectus excavatum	−	−	−	−	Brachytelephalangy	Brachytelephalangy	NA
Investigations										
ALP	NA	NA	High	High	High	High	High	High	High	High
Brain imaging	NA	NA	IVH II, normal structure (brain US)	Normal (CT head)	Normal (MRI brain)	Normal (spinal MRI)	NA	NA	NA	NA
CSF study	NA	NA	NA	NA	NA	NA	NA	NA	NA	NA
Genetics										
Variants^a^	c.[191C > T];[191C > T]^b^	c.[191C > T];[191C > T]^b^	c.[2 T > G];[221G > A]	c.[554G > A];[554G > A]	c.[554G > A];[554G > A]	c.[554G > A];[554G > A]	c.[554G > A];[554G > A]	c.[698C > T];[698C > T]	c.[698C > T];[698C > T]	c.[698C > T];[698C > T]

^a^ Variants listed on transcript NM_001256240.2 other than c.191C > T from Naseer et al. (see [2]).

^b^ Annotated on transcript NM_014489.4.

## 2 METHODS

### 2.1 Patient consent

All procedures followed were in accordance with the ethical standards of the responsible committee on human experimentation (institutional and national) and with the Helsinki Declaration of 1975, as revised in 2000. Informed consent was obtained from the guardians for being included in the study.

### 2.2 Targeted gene sequencing

DNA extracted from blood was subject to library preparation and target enrichment using Agilent's SureSelect Focused Exome +1 (Agilent, http://www.agilent.com). Enriched libraries were sequenced on an Illumina NextSeq 500 (Illumina, https://www.illumina.com/) using a paired‐end 75 base‐pair sequencing protocol. De‐multiplexed data in FASTQ format were analysed using an in‐house developed pipeline, which performed alignment (BWA‐MEM; Burrows Wheeler Aligner v0.7.5‐a: http://bio-bwa.sourceforge.net/), variant calling (FreeBayes v0.9.21; https://github.com/ekg/freebayes) and variant annotation (Alamut‐Batch v1.3.1; http://www.interactive-biosoftware.com/alamut-batch/). Pipeline output was limited to variants within 20 base pairs of the donor and acceptor splice sites of CCDS exons (http://www.ensembl.org/info/genome/genebuild/ccds.html) of the virtual gene panel. Variants were filtered out when present at 2% or greater in ExAC (http://exac.broadinstitute.org/), exome variant server (EVS) (http://evs.gs.washington.edu/EVS/) or 1000 genomes (http://www.internationalgenome.org/) datasets.

### 2.3 Case report

We report the case of a 5 year and 10‐month‐old girl of Afro‐Caribbean descent referred to the Paediatric Metabolic clinic for intellectual disability and speech delay. She was the only child of non‐consanguineous parents, induced at 37 weeks for intrauterine growth retardation secondary to reversed umbilical arterial end‐diastolic flow. The pregnancy was closely monitored due to maternal systemic lupus erythematosus. The family history was otherwise unremarkable with two agnate siblings being healthy.

Parents reported initial concerns regarding low muscle tone, minimal vocalization and delayed skills acquisition from the age of 6 months. Her developmental progress proceeded at a slow pace as she learned to sit unsupported around 1 year of age, walked when 2 years old and started running by 4 years of age. She vocalized using single vowel and consonant sounds. There were persistent feeding difficulties secondary to oro‐pharyngeal incoordination. The patient attended a special needs school where she received physio‐, occupational and speech and language therapy and learned how to communicate in Makaton. Intermittent esotropia and myopic astigmatism was diagnosed at the age of 1 year and a formal assessment of her hearing, at the age of 3 years, was normal.

On clinical examination, her weight was 26.7 kg (98th centile), height of 119 cm (91st centile) and head circumference of 52.3 cm (50th–75th centile).

There were no dysmorphic features or neuro‐cutaneous skin stigmata. Sparse pubic and axillary hair was noted from the age of 4 years. Four limbs hypotonia with positive Gower sign when moving from sitting to standing position was present, suggesting proximal muscles weakness. Tendon reflexes were normal. Her cognitive assessment using Bayley scale 3rd edition showed a 23‐month developmental age. Language assessment (Preschool Language Scales, Edition 5) age was equivalent to 26 months. Severe language impairment affecting both receptive and expressive skills with moderate intellectual disability (DSMV criteria) was confirmed. There was no history of seizures.

Metabolic work‐up including intermediary metabolism, microelements, peroxisomal and lysosomal function, glycosylation, creatine, and purine/pyrimidine metabolites were normal. Initial genetic analysis reported normal array comparative genomic hybridization and fragile X polymerase chain reaction. Routine biochemistry showed persistently elevated alkaline phosphatase 1300–1832 U/L (ref. 150–380) with normal liver function tests. Urinary cortico‐sterol analysis confirmed precocious adrenarche. Wrist X‐ray at the age of 5 years and 8 months found minimal sclerosis of the distal radial metaphysis without other radiographic features of rickets. Bone age was that of 8 years and 11 months.

Cerebrospinal fluid (CSF) neurotransmitter analysis showed low pyridoxal phosphate of 8 mmol/L (ref. 10–37) and 5‐methyltetrahydrofolate (5‐MTHF) of 66 mmol/L (ref. 72–172). Her plasma pyridoxal phosphate was elevated at 190 nmol/L (ref. 15–73) while red cell and plasma folate were normal. In addition, CSF homovanillic acid (HVA) was raised at 611 nmol/L (ref. 71–565) suggesting increased dopamine turnover.

Brain MRI at the age of 6 years was normal. Nerve conduction studies/electromyography, to explore a possible neurogenic cause for oromotor dyspraxia, found no neurogenic changes.

Electroencephalogram (EEG), recorded in wakefulness and sleep, showed a definite, moderate, non‐specific abnormality consisting of brief (1–2 s) runs of 4‐6/s spike–wave complexes over the posterior half of the head, facilitated by eye closure, and possibly by photic stimulation at 16 Hz, (though the phenomenon occurred after eye closure at these times too). During drowsiness, runs lasting 4–6 s were seen without clinical accompaniments. During sleep only isolated 1–2 s bursts of spike–wave complexes were seen over the posterior half of the head, involving the mid‐ but not the anterior temporal electrodes.

The overall clinical picture of persistent hyperphosphatasia in association with mental retardation was further evaluated by next generation sequencing for the GPI anchor defect genes associated with HPMRS. The virtual gene panel was drawn from the OMIM phenotypic series for hyperphosphatasia with mental retardation syndrome (http://omim.org/phenotypicSeries/PS239300, HPMRS1 #239300, HPMRS2 #614749, HPMRS3 #614207, HPMRS4 #615716, HPMRS5 #616025, HPMRS6 # 616809), and consisted of the following genes: *PGAP2*, *PGAP3*, *PIGO*, *PIGV*, *PIGW*. All target bases (CCDS exons and 20 base‐pair flanking intronic regions) were covered by 30 or more reads.

Targeted sequence analysis identified two heterozygous variants in the *PGAP2* gene:

NM_001256240.2 c.103del p.(Leu35Serfs*90) and c.134A > G p.(His45Arg).

The c.103del p.(Leu35Serfs*90) variant is predicted to result in a protein frame‐shift and subsequent premature stop gain, is absent from the gnomAD database (http://gnomad.broadinstitute.org/) and has not previously been reported; it is therefore considered highly likely to be pathogenic (ACMG evidences: PVS1, PM2, PP4_moderate). The c.134A > G p.(His45Arg)variant affects a highly conserved amino acid and is absent from the gnomAD database and has not previously been reported. Analysis of the NGS read data showed these variants to be on opposite alleles (*in trans*) and given the highly specific phenotypic and biochemical support it is therefore considered likely to be pathogenic (ACMG evidences: PM2, PM3, PP4_moderate). Subsequent Sanger confirmation studies confirmed the presence of these variants in the proband, and parental carrier status.

Given the changes observed on EEG and the low 5‐MTHF and pyridoxal phosphate levels in the CSF, despite high plasma pyridoxal phosphate levels, the patient was started on pyridoxine (100 mg twice daily) and folinic acid (15 mg once daily) supplementation at the age of 7 years on which she has continued until today at the age of 11 years. After commencing treatment, the patient continued to make some developmental progress. Her speech improved so that she now communicates in one‐ to two‐word sentences. Her fine motor skills progressed with a more developed pincer grasp. At the age of 8 years she started to experience absence seizures which were well‐controlled on Ethosuximide.

Repeat CSF analysis at the age of 10 years showed normalization of both the 5‐MTHF (78 nmol/L, ref. 46–160) and pyridoxal phosphate level (12 nmol/L, ref. 10–37) as well as the HVA level (388 nmol/L, ref. 71–565).

## 3 DISCUSSION

The patient described here presented with typical characteristics of HPMRS: elevated alkaline phosphatase, intellectual disability, more marked in the language and motor skills and absence seizures. To the best of our knowledge, this is the first patient affected by HPMRS who presented with precocious adrenarche. The patient described is compound heterozygous for variants in the *PGAP2* gene confirming the diagnosis of HPMRS3. Neither the single base deletion c.103del resulting in a protein frameshift with premature stop codon p.(Leu35Serfs*90) nor the missense variant c.134A > G p.(His45Arg) have been reported in the literature previously and expand the genotypic spectrum of this disorder.

Despite description in the literature that pyridoxine supplementation may be beneficial in managing untreatable seizures in HPMRS patients,^2^, ^10^, ^17^, ^18^ this is the first case in which, by means of a CSF study, 5‐MTHF and pyridoxal phosphate levels are demonstrated to be reduced, while normal or elevated in plasma, suggesting a deficiency in neuronal intracellular pyridoxine. Increased dopamine turnover^19^ was demonstrated by raised CSF HVA. A direct correlation between 5‐MTHF and pyridoxal phosphate (PLP) has been described previously.^20^ Reduced serine biosynthesis and serine‐hydroxymethyltransferase activity due to PLP deficiency have been suggested to lead to reduced 5‐MTHF levels in PLP deficient Neuro‐2a cells.^20^ Given that the patient followed a normal diet without biochemical evidence of nutritional deficiencies it is unlikely that the reduced CSF PLP and 5‐MTHF level were due to dietary deficiency, especially when considering that red cell folate and plasma pyridoxal phosphate were normal or elevated. Vitamin B6 and folinic acid supplementation led to normalization of CSF levels in the reported patient suggesting that vitamin B6 and folinic acid should be considered in treating patients with HPMRS. Our patient continued to make developmental progress. Whether some of this was facilitated due to optimizing PLP and 5‐MTHF levels will need to be assessed in larger patient cohorts.

Based on the abovementioned findings and current knowledge, miss‐targeting GPI‐APs could represent a potential pathophysiologic explanation for the CSF abnormalities found in our patient.^1^, ^2^ Alkaline phosphatase is a key enzyme for dephosphorylation of circulating PLP to pyridoxal, the primary vitamin B6 derivative that crosses the blood–brain barrier.^1^ Accordingly, it is known that patients with hypophosphatasia caused by mutations in tissue non‐specific ALP genes, have seizures which can be successfully treated with pyridoxine due to lack of PLP dephosphorylation and secondary lack of pyridoxal to cross the blood brain barrier.^2^ Based on this, a vitamin B6 responsive seizure disorder in hyperphosphatasia with high alkaline phosphatase level may sound paradoxical. Nonetheless, in clinical situations where increased ALP is secondary to bone or liver impairment, elevated enzyme levels are associated with decrease in circulating levels of PLP, that are rapidly dephosphorylated, although seizures are not commonly reported.^21^ For *PIGV* and *PIGO* defects, responsible of HPMRS type 1 and 2, where the ALP is prematurely released within the cell, it can be hypothesized that higher intracellular activity of ALP, and its increased mis localization in neurons,^1^ may result in focal or more widespread CNS deficiency of intracellular PLP, thus contributing to B6‐responsive seizures.^1^, ^2^, ^22^ In HPMRS type 3 though, ALP is prematurely cleaved and released out of the cell.^12^, ^13^ Therefore, the mechanism leading to low CSF PLP in HPMRS 3 remains unclear. Nevertheless, we can hypothesize at least two mechanisms: either overall decreased levels of PLP secondary to rapid dephosphorylation in a context of increased ALP activity or intracellular re‐localization of ALP secondary to an extracellular excess, due to its premature and excessive cleavage from the cellular surface. Certainly, the cause of low PLP in CSF in PGAP2 mutation cause defects warrants further studies.

In conclusion, the case of HPMRS3 discussed here suggests that low PLP and 5‐MTHFR levels in the CSF may be a characteristic of HPMRS that is amenable to therapeutic correction. Further studies on larger patient cohorts are required to confirm this hypothesis and assess the effect of pyridoxine and folinic acid supplementation on clinical outcome.

## AUTHOR CONTRIBUTIONS

MM and EM reviewed the literature and wrote the manuscript with support from KT. KT and TC supported in writing the manuscript and reviewed the molecular data. SB conceived the original idea and supervised the report.

## CONFLICT OF INTEREST

Martina Messina declares that she has no conflict of interest. Emanuela Manea declares that she has no conflict of interest. Thomas Cullup declares that he has no conflict of interest. Karin Tuschl declares that she has no conflict of interest. Spyros Batzios declares that he has no conflict of interest. No competing interests were disclosed.

## PATIENT CONSENT STATEMENT

Informed consent was obtained from patient's parent for being included in the study.

## ANIMAL RIGHTS

This article does not contain any studies with human or animal subjects performed by any of the authors.

## ETHICS STATEMENT

There was no requirement of ethical approval as this is a case report.

## Data Availability

My manuscript has no associated data.
